# A 14-Year Italian Experience in DM2 Genetic Testing: Frequency and Distribution of Normal and Premutated *CNBP* Alleles

**DOI:** 10.3389/fgene.2021.668094

**Published:** 2021-06-21

**Authors:** Annalisa Botta, Virginia Veronica Visconti, Luana Fontana, Paola Bisceglia, Mario Bengala, Roberto Massa, Ilaria Bagni, Rosanna Cardani, Federica Sangiuolo, Giovanni Meola, Giovanni Antonini, Antonio Petrucci, Elena Pegoraro, Maria Rosaria D’Apice, Giuseppe Novelli

**Affiliations:** ^1^Medical Genetics Section, Department of Biomedicine and Prevention, University of Rome Tor Vergata, Rome, Italy; ^2^Laboratory of Medical Genetics, Tor Vergata Hospital, Rome, Italy; ^3^Research Laboratory, Complex Structure of Geriatrics, Fondazione IRCCS Casa Sollievo della Sofferenza, San Giovanni Rotondo, Italy; ^4^Neuromuscular Disease Unit, Department of Systems Medicine, University of Rome Tor Vergata, Rome, Italy; ^5^BioCor Biobank, UOC SMEL-1 of Clinical Pathology, IRCCS-Policlinico San Donato, Milan, Italy; ^6^Department of Biomedical Sciences for Health, University of Milan, Milan, Italy; ^7^Department of Neurorehabilitation Sciences, Casa di Cura del Policlinico, Milan, Italy; ^8^Neuromuscular and Rare Disease Center, Department of Neuroscience, Mental Health and Sensory Organs (NESMOS), Sant’Andrea Hospital, Sapienza University of Rome, Rome, Italy; ^9^Center for Neuromuscular and Neurological Rare Diseases, S. Camillo Forlanini Hospital, Rome, Italy; ^10^Department of Neuroscience, University of Padua, Padua, Italy

**Keywords:** myotonic dystrophy type 2, *CNBP* premutations, alleles distribution, DM2 genetic testing, penetrance

## Abstract

Myotonic dystrophy type 2 (DM2) is a multisystemic disorder caused by a (CCTG)_*n*_ in intron 1 of the *CNBP* gene. The CCTG repeat tract is part of a complex (TG)_*v*_(TCTG)_*w*_(CCTG)_*x*_(NCTG)_*y*_(CCTG)_*z*_ motif generally interrupted in *CNBP* healthy range alleles. Here we report our 14-year experience of DM2 postnatal genetic testing in a total of 570 individuals. The DM2 locus has been analyzed by a combination of SR-PCR, TP-PCR, LR-PCR, and Sanger sequencing of *CNBP* alleles. DM2 molecular diagnosis has been confirmed in 187/570 samples analyzed (32.8%) and is mainly associated with the presence of myotonia in patients. This set of *CNBP* alleles showed unimodal distribution with 25 different alleles ranging from 108 to 168 bp, in accordance with previous studies on European populations. The most frequent *CNBP* alleles consisted of 138, 134, 140, and 136 bps with an overall locus heterozygosity of 90%. Sequencing of 103 unexpanded *CNBP* alleles in DM2-positive patients revealed that (CCTG)_5_(NCTG)_3_(CCTG)_7_ and (CCTG)_6_(NCTG)_3_(CCTG)_7_ are the most common interruption motifs. We also characterized five *CNBP* premutated alleles with (CCTG)_*n*_ repetitions from *n* = 36 to *n* = 53. However, the molecular and clinical consequences in our cohort of samples are not unequivocal. Data that emerged from this study are representative of the Italian population and are useful tools for National and European centers offering DM2 genetic testing and counseling.

## Introduction

Myotonic dystrophy type 2 (DM2; MIM#602668) is an autosomal dominant multisystemic disorder caused by a (CCTG)_*n*_ repeat expansion in intron 1 of *CNBP* gene (previously *ZNF9*) on chromosome 3q21.3 (Liquori et al., 2001). DM2 is characterized by progressive proximal muscle weakness, myotonia, myalgia, calf hypertrophy, and multiorgan involvement with cataract, cardiac conduction defects, and endocrine disorders (Meola and Cardani, 2015; Montagnese et al., 2017). The clinical phenotype typically arises in the adulthood, and to date, no congenital form of the disease has been reported (Meola et al., 2017). The true prevalence of DM2 is still uncertain since DM2 is almost under diagnosed; nevertheless, clinical experience indicates that DM2 is roughly fivefold less common than myotonic dystrophy type 1 (DM1; MIM# # 160900), at least in the Mediterranean area. DM2 mutations have been identified mainly in North Eastern Europe, where the DM2 expansion only occurs on a specific chromosomal haplotype, suggesting the occurrence of a predisposing mutation in a common ancestral founder (Bachinski et al., 2003; Liquori et al., 2003; Coenen et al., 2011; Meola and Cardani, 2017). The CCTG repeat tract is part of a complex (TG)_*v*_(TCTG)_*w*_(CCTG)_*x*_ motif, which is generally interrupted in healthy range alleles by one or more GCTG, TCTG, or ACTG (NCTG) motifs resulting in more stability (Radvanszky et al., 2013). Consequently, the combined repeat tract in those healthy range alleles can be described by five distinct repetitive motifs (TG)_*v*_(TCTG)_*w*_(CCTG)_*x*_(NCTG)_*y*_(CCTG)_*z*_ (Bachinski et al., 2009). Normal alleles have <25 copies of the CCTG repeat tract, whereas expanded alleles have as many as 75–11,000 copies (Thornton, 2014). Alleles between 27 and 74 CCTG units (called gray area) are very rare, and their clinical significance is still unclear and subject of discussion in the scientific community. In DM2 patients, the interval of CCTG repeat units is extremely wide, ranging from 75 to over 11,000 units with a mean of 5,000 (Turner and Hilton-Jones, 2010). Unstable expanded alleles have an uninterrupted CCTG tract which favors unusual secondary structure more susceptible to strand slippage and unequal crossing over during cell division (Bachinski et al., 2009; Guo and Lam, 2016). The CCTG repeat size increases with age with a high level of somatic mosaicism; however, different from DM1, no significant correlation has been observed between CCTG repeat size and age of onset or other measures of disease severity (e.g., muscle weakness and age of cataract extraction) (Day et al., 2003). More interestingly, on transmission to the next generation, *CNBP* repeat length sometimes tends to decrease dramatically, without significant differences determined by the parental origin of the mutation (Day et al., 2003). The pathogenic mechanism of DM2 is an RNA gain of function toxicity leading to the formation of CCUG-containing ribonuclear foci and spliceopathy mediated mainly by the sequestration of MBNL1 protein (Cardani et al., 2009; Meola and Cardani, 2015). The molecular diagnostic protocol of DM2 is a challenging approach because of the CCTG expansion and the high level of somatic mosaicism. The initial step is the short-range PCR (SR-PCR), which allows excluding the DM2 diagnosis in the case of two alleles in the normal range. If only one allele is visible, CCTG expansion can be detected either with long-range PCR (LR-PCR) or tetraplet-primed PCR (TP-PCR) leading to about 99% of detection rate (Kamsteeg et al., 2012). The interpretation of the molecular data can be complicated by the polymorphic variability of *CNBP* normal alleles and by the length of the CCTG expansion, which can be only determined with classical Southern blot on digested genomic DNA. The molecular characterization and the distribution of the complex repeated motif and allele frequency of *CNBP* alleles in the normal population has been already reported only in German, Slovak, and American populations (Bachinski et al., 2009; Radvanszky et al., 2013; Mahyera et al., 2018). In the present work, we report our 14-year experience (from 2007 to 2020) of DM2 postnatal genetic testing in a total of 570 individuals from the Italian population. Main clinical indications in DM2 positive genetic testing, allele length, frequency, and distribution of *CNBP* normal alleles and detailed composition of the complex (TG)_*v*_(TCTG)_*w*_(CCTG)_*x*_(NCTG)_*y*_(CCTG)_*z*_ are given. Furthermore, we also described at the clinical and molecular levels five individuals carrying *CNBP* unstable premutated alleles with the intent to provide useful information in DM2 genetic counseling.

## Materials and Methods

### Individuals Included in This Study

The enrollment of the subjects described in this study was approved by the Institutional Review Board of Policlinico Tor Vergata (document no. 232/19). All individuals were referred to our center for DM2 genetic testing, and informed consents have been obtained from each participant. All individuals were of Caucasian origin from Italy. Muscle biopsies used for this study have been collected in Policlinico San Donato after receiving written informed consent from the patients (study authorized by the Institutional Ethics Committee ASL MI2-Melegnano via VIII Giugno, Milan).

### *CNBP* Fragment Analysis Data

DNA was extracted from peripheral blood using the EZ1 Advanced XL Robotic workstation for automated purification of nucleic acids (QIAGEN, Germany). The molecular characterization of the DM2 mutation has been carried out using a combination of short-range PCR (SR-PCR), tetraplet-primed PCR (TP-PCR) and long-range PCR (LR-PCR), as described (Botta et al., 2006; Kamsteeg et al., 2012). SR-PCR enabled us to assess the length of the complex motif, and up to 40 CCTG repeats can be amplified using this method. DNA region was amplified with the following PCR pair primers: CL3N58D-F (5′-GGCCTTATAACCATGCAAA-3′) and CL3N58D-R (5′-CCTAGGGGACAAAGTGAG-3′). Amplification was carried out in 30 μl volume, containing: 50 ng genomic DNA, PCR reaction buffer (5×), 25 mM MgCl_2_, 1.25 mM dNTPs, 70 pmol of each primer, and 1 U of TaKaRa Taq polymerase (Takara Bio). The PCR cycle program consists of the following amplification conditions: 94°C for 3 min, 30 cycles of denaturation at 94°C for 1 min, annealing at 58°C for 40 s, and extension at 72°C for 40 s. A final elongation was carried out at 72°C for 5 min. Finally, capillary electrophoresis was carried out in order to determine the length of the *CNBP* unexpanded alleles in DM2-negative individuals. The statistical analysis of the frequency and distribution of the different 756 *CNBP* normal allele lengths characterized was done with MS Excel (Office Suite 2016).

### Sequencing of *CNBP* Normal and Premutated Alleles

Sanger sequencing was performed to characterize *CNBP* repeat motif compositions of non-DM2 alleles. To simplify the analysis, PCR products were amplified from affected offspring who inherited the normal haplotype of interest, as only a single allele within the normal range would be amplified. SR-PCR products were purified and sequencing directly using BigDye Terminator v1.1 Cycle Sequencing Kit and visualized by capillary electrophoresis on an Applied Biosystems 3130xl Genetic Analyzer. Sequencing conditions were the following: 96°C for 1 min, 25 cycles of denaturation at 96°C for 10 s, annealing at 50°C for 5 s and extension at 60°C for 4 min. Data were analyzed with the software Sequencing Analysis v5.2 (Applied Biosystem).

### Muscle Histopathology and RNA FISH/MBNL1 Immunofluorescence Analyses

Human muscle biopsies from biceps brachii muscle were taken under sterile conditions. Muscle samples were trimmed of blood vessels, fat, and connective tissues and fresh-frozen in isopentane cooled in liquid nitrogen. Histopathological analysis was performed on serial sections (8 μm) processed for routine histological or histochemical stainings. A standard myofibrillar ATPase staining protocol was used after preincubation at pH 4.3, 4.6, and 10.4. The most typical alterations, such as nuclear clump fibers (i.e., aggregates of myonuclei with a thin rim of cytoplasm), nuclear centralization, and fiber size variability, with type II atrophy, were evaluated on serial muscle sections. Fluorescence *in situ* hybridization was performed on muscle frozen sections using a (CAGG)_5_ probe as previously reported by Cardani et al. (2009) to verify the presence of ribonuclear inclusions and their colocalization with MBNL1 protein.

### Study of Alternative Splicing

Frozen muscle samples were practiced for the extraction of total RNA using TRIzol reagent (Gibco BRL, Gaithersburg, MD, United States), and 1 μg of RNA was reverse transcribed according to the cDNA protocol of the High Capacity cDNA Archive kit (Applied Biosystems, Foster City, CA, United States). Splicing pattern profile of the *IR*, *CLCN1*, and *MBNL1* genes was carried out as described (Nakamori et al., 2013). Total PCR products, obtained within the linear range of amplification, were electrophoresed on 2.5% agarose gel. Quantitative analysis of the amplified products was performed using SYBR Green II stained gels (Perkin-Elmer Life Science, Boston, MA, United States) scanned on a fluorimager 595 (Amersham Biosciences, Buckinghamshire, United Kingdom). The intensity of each band and the fraction of abnormally (or pathologically) spliced (AS) isoforms (AS-isoforms/total) were quantified by densitometry using ImageQuant software. Control of the RT-PCR reaction was based on the expression level of the glucose phosphate isomerase housekeeping gene (*GPI*) and all amplifications have been carried out in triplicate using independent cDNA samples.

## Results

### Sociodemographic Characteristics of DM2 Patients and Major Clinical Indications for DM2 Genetic Testing

**FIGURE 1:**
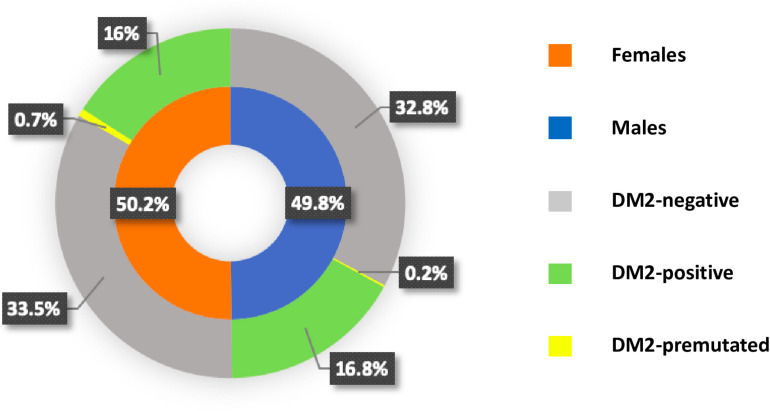
Pie chart showing the frequency of myotonic dystrophy type 2 (DM2)-positive, DM2-negative, and DM2-premutated with sex distribution in each category.

**FIGURE 2:**
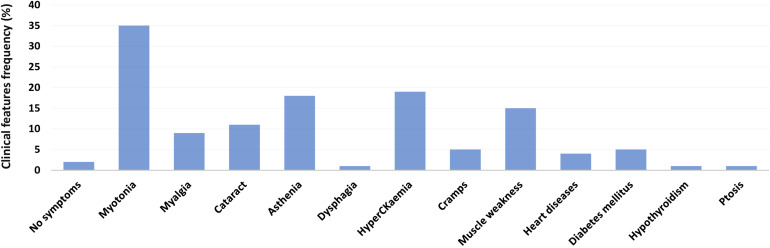
Main clinical features of genetically confirmed DM2 patients (*n* = 187).

The Medical Genetics Unit at Tor Vergata Hospital is one of the main reference diagnostic labs for myotonic dystrophies in Italy. From 2007 to 2020, a total of 570 DM2 molecular analyses have been performed (286 females and 284 males) (Table 1). The main clinical indications for DM2 genetic testing were myotonia (28%), familiarity for the disease (24%), asthenia (20%), hyperCKaemia (18%), muscle weakness (17%), myalgia (15%), and cataract (6%). After DM2 genetic testing, only 187 individuals (33%) showed the presence of a (CCTG)_*n*_ expansion in the *CNBP* gene. Among 187 DM2 confirmed patients, 96 were males (51%) and 91 were females (49%), with a mean disease age onset of 49 ± 16.9 years without a significant difference between sexes (*p* = 0.2) (Figure 1). The low percentage of DM2-positive test is in accordance with a data reported by Mahyera et al. (2018) and demonstrates that most clinicians still do not easily recognize the cardinal features of the DM2 disease. A positive family history was referred by 70/187 DM2-confirmed patients (37%); the familiarity was considered “positive” if patients reported of family members diagnosed with DM2. The confirmation of the DM2 mutation is mainly associated with the presence of myotonia (35%), hyperCKaemia (19%), asthenia (18%), muscle weakness (15%), and cataract (11%) in our cohort. On the contrary, cardiac, and endocrine dysfunctions are not specifically associated with the DM2 phenotype being present in less than 5% of DM2-positive tests (Figure 2). However, it is important to point out that, given the heterogeneity of submitters from all over the country, some clinical information may be missed or incomplete. In DM2 families where the transmission of the expansion could be determined (*n* = 62), no differences have been found between the parental origin of the DM2 mutation.

**TABLE 1:** DM2 genetic tests requested to our lab in the period between 2007 and 2020.

Year	Cohort (*n* = 570)	Males (*n* = 284)	Females (*n* = 286)	DM2 positive (*n* = 187)
2020	30	14	16	7
2019	52	27	25	22
2018	56	22	34	18
2017	57	34	23	11
2016	63	27	36	23
2015	49	19	30	14
2014	40	20	20	14
2013	20	7	13	8
2012	39	21	18	17
2011	42	23	19	13
2010	35	19	16	12
2009	34	21	13	11
2008	40	22	18	14
2007	13	8	5	3

### Frequency and Distribution of *CNBP* Healthy Range Alleles

**FIGURE 3:**
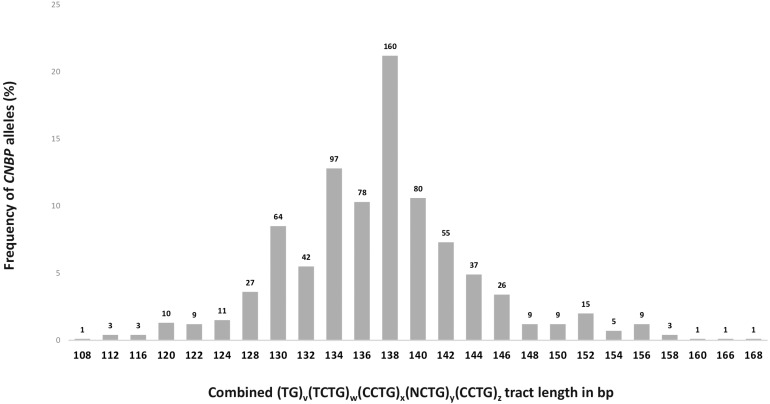
Frequency and distribution of *CNBP* healthy range alleles in DM2-negative Italian individuals (*n* = 378). Absolute number of alleles has been reported above the bars in the graph (total number of alleles = 756).

To define the polymorphic spectrum of the (TG)_*v*_ (TCTG_)w_(CCTG)_*x*_(NCTG)_*y*_(CCTG)_*z*_ repeated motif in the Italian population, we analyzed the distribution of *CNBP* alleles in non-DM2 chromosomes. Fragment analysis by capillary electrophoresis was used to calculate the length of unexpanded *CNBP* normal alleles in 378 DM2-negative Italian individuals. The combined repeat tract length was plotted against the allele frequency. In this cohort of samples, a unimodal distribution of 25 different *CNBP* alleles length is shown, ranging from 108 to 168 bps (Figure 3). The most frequent alleles in the Italian population consisted of 138 (21%), 134 (13%), 140 (11%), and 136 (10%), and these data are in accordance with the data reported in the Slovak and German populations (Radvanszky et al., 2013; Mahyera et al., 2018). The combined use of SR-PCR and LR-PCR in DM2-negative probands revealed a homozygosity frequency for the *CNBP* locus of 90%, according to published data (Liquori et al., 2001; Mahyera et al., 2018).

### Structure and Composition of the (TG)_*v*_(TCTG)_*W*_(CCTG)_*X*_(NCTG)_*y*_(CCTG)_*z*_ Repeated Motif in *CNBP* Normal Alleles

**FIGURE 4:**
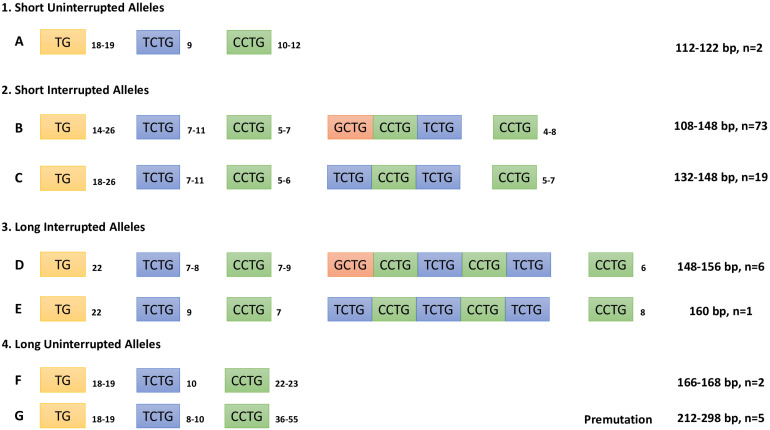
Composition of the combined repeat tract (TG)_*v*_(TCTG)_*w*_(CCTG)_*x*_(NCTG)_*y*_(CCTG)_*z*_ of *CNBP* non-expanded alleles determined by Sanger sequencing analysis. Repeat tract length in bp and number of alleles characterized are also indicated.

To determine the composition of the complex repeated tract in the *CNBP* gene, we sequenced *CNBP* normal alleles in 103 non-DM2-chromosomes: 87 derived from DM2-positive patients with full expansions and 16 from DM2-negative probands. Five additional DNA samples belonging to patients with mild muscular phenotype requesting DM2 genetic testing have also been analyzed. At least one sample in each allele length class has been sequenced; for the most frequent alleles, multiple samples have been characterized (138 bp, *n* = 20; 134 bp, *n* = 14; 140 bp, *n* = 9; and 136 bp, *n* = 14). The composition of the *CNBP* healthy range alleles is summarized in Figure 4. Results allow to distinguish four allelic classes: (1) short uninterrupted alleles with (CCTG)_10__–__12_ (2) short interrupted alleles with (CCTG)_9__–__15_ containing three interruption motifs, (3) long interrupted alleles with (CCTG)_13__–__17_ containing five interruption motifs, and 4) long uninterrupted alleles with (CCTG)_22__–__55_ including five DM2 premutations. Short uninterrupted alleles with (CCTG)_10__–__12_ are very rare alleles representing only 2% of normal *CNBP* chromosomes in our population, in accordance with published data (Radvanszky et al., 2013). *CNBP* interrupted alleles were divided into long and short classes depending on the length of the interruption located within the (CCTG)_*n*_ array: short alleles with (CCTG)_9__–__15_ interrupted by three tetraplets (NCTG)_3_: T/GCTG-CCTG-TCTG and longer alleles containing (CCTG)_13__–__15_ interrupted by domains containing five tetraplets (NCTG)_5_: T/GCTG-CCTG-TCTG-CCTG-TCTG repetitions. Interestingly, the most frequent *CNBP* alleles have a (CCTG)_*x*_(NCTG)_3_(CCTG)_*z*_ structure (92.9%), whereas only 7.1% contains (CCTG)_*x*_(NCTG)_5_(CCTG)_*z*_ motifs. The longest 168-bp allele contains no interruptions of the (CCTG) tract (*y* = 0) with a (TG)_18_(TCTG)_10_(CCTG)_23_ structure and is identical to the longest *CNBP* allele reported in German non-DM2-chromosomes. The shortest 108-bp allele has been also sequenced resulting with the following repeated tract (TG)_16_(TCTG)_7_(CCTG)_5_(NCTG)_3_(CCTG)_4_. Analysis of the repeated motifs within the interruption tract shows that the most common combinations are represented by (CCTG)_5_(NCTG)_3_(CCTG)_7_ and (CCTG)_6_(NCTG)_3_(CCTG)_7_, with a frequency of 62.1% and 20.4%, respectively. Furthermore, by analyzing the whole repeated stretch (TG)_*v*_(TCTG)_*W*_(CCTG)_*X*_(NCTG)_*y*_(CCTG)_*z*_, we noticed that both (TG)_21_(TCTG)_9_(CCTG)_5_(NCTG)_3_(CCTG)_7_ and (TG)_17_(TCTG)_9_(CCTG)_5_(NCTG)_3_(CCTG)_7_ motif combinations (138 p and 130 bp alleles, respectively) were present in 8.7% of the sequenced alleles. In contrast to the length of the whole (TG)_*v*_(TCTG)_*w*_(CCTG)_*x*_(NCTG)_*y*_(CCTG)_*z*_ motif, the (CCTG)_*x*_ tract alone was found to be less polymorphic, with 16 different alleles identified in our cohort of samples. On the other hand, the (TG)_*v*_ and the (TCTG)_*w*_ tracts upstream of the CCTG array are highly polymorphic within the range of v and w tract, showing hypervariability of the (TG)_*v*_ and (TCTG)_*w*_ tracts with *v* = 14–26 and *w* = 7–11. Given the extreme variability in the sizes of the (TG) and (TCTG) different subunits, the precise length of the pathogenic CCTG unit within this repeat can only be determined by DNA sequencing when DM2 genetic testing is performed. Interestingly, we identified the presence of an uninterrupted (CCTG)_*n*_ tract below 26 repetitions, which is considered the upper threshold for stable *CNBP* alleles, in 4% of non-DM2 chromosomes Overall, we can consider our data representative of the genetic variability associated with the DM2 locus in the Italian population. The general structure of the *CNBP* repeated motif in non-DM2 chromosomes is the following: (TG)_14__–__26_(TCTG)_7__–__11_(CCTG)_5__–__9_(NCTG)_3__–__5_(CCTG)_4__–__8_.

### *CNBP* Premutated Alleles Are Unstable and Have Uncertain Functional and Clinical Consequences

**FIGURE 5:**
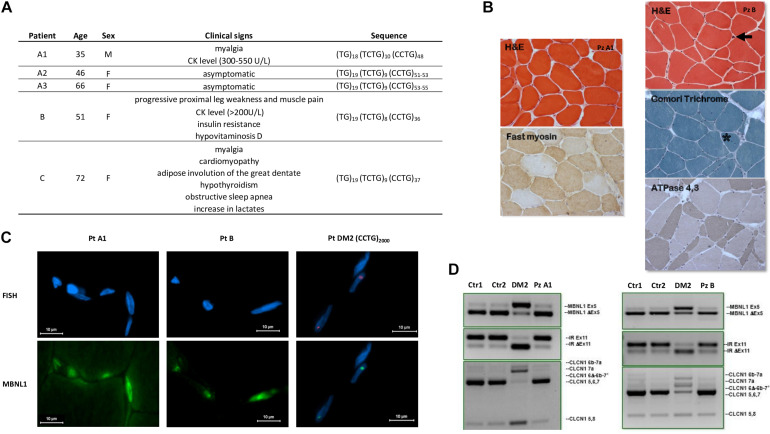
Patients with *CNBP* premutated alleles. **(A)** Clinical characteristics of DM2 premutated patients and sequence of the uninterrupted *CNBP* alleles. **(B)** Left: Patient A1, Biceps brachii muscle biopsy performed at the age of 16 shows only a slight fiber size variability both of type I and II fibers (200×). Right: Patient B, Biceps brachii muscle biopsy performed at the age of 51 shows an increase of fiber size variability, nuclear clumps (arrow), central nuclei (asterisk) and type I (200×). **(C)** FISH + MBNL1 immunofluorescence of patient A1 and B shows no nuclear accumulation of *CNBP* mutant RNA or of MBNL1 as instead observable in a DM2 patient with (CCTG)_2000_ repetitions. **(D)** Similarly to healthy individuals (Ctr1 and Ctr2), no alterations of alternative splicing of *MBNL1*, *IR*, and *CLCN1* genes have been evidenced by RT-PCR analysis compared with a DM2 patient, used as positive control.

During our diagnostic procedure, we characterized also five DM2-negative patients carrying *CNBP* premutated alleles from individuals referred to our centers for DM2 genetic testing (Figure 5A). Patient A1 is a 35-year-old male who came to our attention because he suffered from myalgia since the age of 16, and no other clinical signs have been reported. EMG did not reveal myotonic discharges with either myopathic or neurogenic changes in vastus lateral muscle. The CK level was mildly elevated with values ranging between 300 and 550 U/L. SR-PCR and Sanger sequencing showed that he is a carrier of a (CCTG)_48_ *CNBP* uninterrupted allele. The asymptomatic sister (A2), 46 years of age, carries a (CCTG)_51__–__53_ *CNBP* uninterrupted allele, and both premutations were inherited from the 66-year-old asymptomatic mother (A3) harboring (CCTG)_53__–__55_ repetitions. Patient B is a 51-year-old woman, carrying a (CCTG)_36_ *CNBP* uninterrupted allele, whose clinical signs reflect progressive proximal leg weakness and muscle pain, iperCKemia (>200 U/L), no clinical or EMG myotonia, insulin resistance, and hypovitaminosis D. Her father died of respiratory insufficiency at 69 years old and was reported to be affected by a not defined muscular dystrophy. Finally, patient C is a 72-year-old woman, carrying a (CCTG)_37_ *CNBP* uninterrupted allele, who has been suffering from myalgia since the age of 9. She has cardiomyopathy and is a carrier of PM. In her family history, premature deaths are also reported. In addition, she showed adipose involution of the great dentate, no EMG myotonia, hypothyroidism, obstructive sleep apnea, normal CK level, and a slight increase in lactates. Unfortunately, biceps brachii muscle biopsies were available for histological and molecular analyses only for patients A1 and B. Muscle biopsy from patient A1 showed only a slight fiber size variability both of types I and II fibers (Figure 5B). Analysis of muscle tissue from patient B revealed an increase in fiber size variability, nuclear clumps, central nuclei, and types I and II fiber atrophy (Figure 5B). RNA FISH and MBNL1 immunofluorescence analyses showed no nuclear accumulation of *CNBP* mutant RNA of MBNL1 protein in myofibers from both patients (Figure 5C). Similar to healthy individuals, no alterations in alternative splicing of muscle blind-like 1 (*MBNL1*), insulin receptor (*IR*), and chloride voltage-gated channel 1 (*CLCN1*) genes have been evidenced by RT-PCR analysis performed on RNA extracted from muscle biopsies (Figure 5D).

## Discussion

In this study, we report our 14-year experience in DM2 genetic testing as one of the main Italian reference centers for the molecular diagnosis of myotonic dystrophies. The prevalence of DM2 likely varies by population: DM2 is rare in the Japanese population (Matsuura et al., 2012), and in the United States, clinical experience suggests that DM2 is roughly fivefold less common than DM1 (Thornton, 2014; Meola and Cardani, 2015). Data about the prevalence of DM2 in Europe are limited except for the German and Finland populations, where it may be like that reported for DM1 (Suominen et al., 2011; Mahyera et al., 2018). The only study performed in Italy, so far, includes the Rome province with an estimated prevalence of DM2, 10% that of DM1 (Vanacore et al., 2016). Unfortunately, we cannot calculate with accuracy the true prevalence of the DM2 disease in Italy because our data do not include the totality of DM2 patients overall the country. Nevertheless, since we are one of the main national diagnostic labs for myotonic dystrophies, we can estimate that the DM2 tests performed represent about 50% of all DM2 analyses in Italy. This allows us to calculate an estimated prevalence of DM2 in the Italian population that is about 10% that of DM1, in accordance with our previous published data (Vanacore et al., 2016). Moreover, additional important considerations are possible. The first is that we observed a very low percentage of DM2-positive test (33%) compared with the number of samples sent to our lab with a clinical suspicion of DM2. This positivity rate is also lower than what we have experienced in DM1 genetic testing (45% from our unpublished data) and suggests that clinical indications for DM2 molecular diagnosis are generally less defined than in DM1. This conclusion fully agrees with what was reported by Mahyera et al. (2018) for one of the main DM diagnostic labs in Germany and demonstrates how most clinicians still do not easily recognize the cardinal features of the DM2 disease probably due to its rather aspecific and late onset. Consequently, the average delay between the onset of symptoms and the genetic confirmation of DM2 is 14 years (Hilbert et al., 2013). Patients with DM2 usually come to medical attention because of myotonia, prominent in distal limb muscles, however, this symptom is present in less than 50% of subjects. Other symptoms such as cardiac and endocrine dysfunctions are not specifically associated with the DM2 phenotype being present in less than 5% of DM2-positive tests. Recent studies indicate that, unlike DM1, either *CLCN1* or *SCN4A* acts as genetic modifiers in DM2 patients since mutations/polymorphisms in these genes may contribute to exaggerated phenotype with more severe muscle stiffness and myotonia (Suominen et al., 2008; Cardani et al., 2012; Bugiardini et al., 2015; Peddareddygari et al., 2016; Binda et al., 2018). This suggests that DM2 patients with co-segregating *CLCN1* or *SCN4A* genetic variants could be more easily identified than DM2 patients without the modifier alleles, and consequently that the majority of patients remain undiagnosed even in clinical centers with considerable experience with DM2. Among 190 DM2 confirmed patients, we did not observe a different distribution of the disease symptoms and age of onset between males and females. Gender differences have been identified with a tendency toward the worsening of symptoms in females (Montagnese et al., 2017). However, we should point out that, different from the study of Montagnese et al., our work is not focused to provide a detailed clinical characterization of DM2 patients. Most of the clinical information has been collected retrospectively over time from different Italian centers with no follow-up of patients; it is therefore possible that some clinical data may be incomplete, underestimated, or missed at all. For this reason, a gender and age influence on the DM2 phenotype cannot be ruled out on the basis of our data. Comparison with the complex (TG)_*v*_(TCTG)_*w*_(CCTG)_*x*_(NCTG)_*y*_(CCTG)_*z*_ repeat tract length in the Italian population vs. the German and Slovak populations showed that the distribution and the size of the *CNBP* repeat tract is conserved among different European populations (Radvanszky et al., 2013; Mahyera et al., 2018). Normal *CNBP* alleles from DM2-negative probands showed a unimodal distribution with allele sizes ranging from 108 to 168 bps, including very rare alleles bigger than 158 bp in the larger extreme end of the distribution. The range of *CNBP* normal alleles was 118–156 bp in a cohort of 95 proband in the Slovak population and 102–166 in the German population, where a cohort of 739 probands, closer in size to the one reported here, has been analyzed. The four most frequent *CNBP* allele among Italians consisted of 138 (21%), 134 (13%), 136 (11%), and 140 (10%) bps. Frequencies of the 138-, 134-, and 140-bp alleles are identical to the German population, whereas the 142-bp allele, which was found to be the third most frequent allele in Germany and the second most frequent allele in Slovenia, was at fifth place among the Italians (11 and 13% vs. 7% in Italians). However, it should be pointed out that these studies analyzed different study cohorts consisting of normal individuals from the general population (Radvanszky et al., 2013) or DM2-negative probands showing DM2-like symptoms (Mahyera et al., 2018 and our study). In normal-sized alleles from DM2 patients, the CCTG repeat tract is usually found to be interrupted by one or two tetraplets (NCTG)_*n*_, which modulate the genetic stability of the locus. However, as the difference between normal alleles (repeats to 26 CCTG units) and disease-associated alleles (75 units and more, with a mean of 5,000) is usually evident, exact sizing is not routinely performed. The variability in the sizes of the different subunits of the complex repeat, (TG)_*v*_(TCTG)_*w*_(CCTG)_*x*_, in the *CNBP* gene complicates the determination of the precise number of the CCTG unit within this repeat, which can only be determined by DNA sequencing. Our characterization of *CNBP* normal alleles confirms the highly polymorphic nature of the (TG)_*v*_(TCTG)_*w*_ repeated tract compared with the whole (TG)_*v*_(TCTG)_*w*_(CCTG)_*x*_ motif. Indeed, a great variability of these repeat units was found, between (TG)_14__–__26_ and (TCTG)_7__–__11_, which is not proportional to the increase in allelic size. Interestingly, sequencing analysis allowed us to identify two short uninterrupted alleles containing, respectively, (CCTG)_10_ and (CCTG)_12_ repetitions. These rare alleles found in our study cohort, represent only 2% of normal *CNBP* chromosomes, in accordance with previous analysis (Radvanszky et al., 2013). The existence of *CNBP* alleles with uninterrupted CCTG repeated tract has been reported for the first time by Liquori et al. (2003) who described a 136-bp allele containing (CCTG)_20_, thus, considering the 20 CCTG uninterrupted repeated as the shortest possible DM2 premutation allele. A few years later, Bachinski et al. (2009) identified 4 of 44 alleles with uninterrupted (CCTG)_24__–__32_. More recently, in the Slovak population, Radvanszky described six additional *CNBP* uninterrupted alleles containing up to 70 repeats in a large cohort of about 500 individuals. Interestingly, short alleles containing (CCTG)_12__–__14_ among the uninterrupted alleles have been reported, according to our data (Radvanszky et al., 2013). Finally, Mahyera et al. (2018) detected the presence of a 168-bp healthy allele containing (CCTG)_23_ in a total of 34 alleles in DM2-positive patients. There are at least two main still unsolved questions regarding *CNBP* uninterrupted alleles below the “70 CCTG threshold”: their genetic instability (either somatic or germinal) and their clinical significance. The international guidelines for the DM2 molecular diagnosis consider non-pathogenic *CNBP* alleles as those having up to (CCTG)_26_ with one or more interruptions (Bachinski et al., 2009; Kamsteeg et al., 2012), whereas disease-associated alleles contain 75 units and more uninterrupted CCTG units. No information is given on the range and penetrance of premutated *CNBP* alleles comprised of between 26 and 75 CCTG units. What do we know about these premutated alleles from literature? The paper from Bachinski and colleagues demonstrated that uninterrupted (CCTG)_22__–__33_ alleles are unstable and represent a premutation allele pool for DM2 full mutations. Mahyera et al. (2018) also reported two patients harboring (CCTG)_55_ and (CCTG)_30_ *CNBP* alleles with a DM2-like phenotype and (mild myopathy), respectively. (CCTG)70 and (CCTG) > 70 *CNBP* alleles were found in individuals belonging to families with genetically proven Huntington disease (HD); unfortunately, no detailed clinical information was available from these patients (Radvanszky et al., 2013). Our study enlarges the cohort of *CNBP* premutated individuals described so far. During our diagnostic procedure, we characterized five *CNBP* premutated alleles ranging from 36 to 55 uninterrupted CCTG repetitions. The analysis of our two familial cases confirmed that *CNBP* alleles containing more than 50 uninterrupted CCTG are unstable, as previously described. However, the clinical consequences are uncertain leading to only mild myopathy and hyperCKaemia in one patient, whereas his mother is asymptomatic by the age of 66. On the other side, one patient (patient C) carrying (CCTG)_37_ repetitions shows a more complex phenotype with myalgia, hyperthyroidism, and familiar cardiomyopathy. However, no additional genetic tests have been performed to exclude the presence of other genetic causes associated with the observed clinical phenotype. Histological examinations of muscle tissues, available only for two of these premutated individuals, showed mild muscular pathogenic signs with atrophy of both types I and II fibers, which have not been specifically associated with DM2 where high rates of atrophic and hypertrophic type 2 fibers were observed (Pisani et al., 2008). Moreover, no molecular hallmarks of DM (*IR*, *MBNL1*, *CLCN1* splicing alterations, and CCUG-containing foci accumulation) have been detected in muscle tissues. Our data argue in favor of an incomplete penetrance for uninterrupted *CNBP* alleles in the range of (CCTG)_36__–__55_ repetitions. Taken together, our published studies indicated that there is no valid “cut-off” to distinguish *CNBP* alleles in the clinical range from those in the non-pathological range. Rather, there is an overlap of repeat sizes, some of which will be associated with mild disease expression, while others will not, depending upon still unknown modifying factors whose identification may represent a fruitful approach to defining therapeutic strategies in DM2. To conclude, our findings extend current knowledge concerning the polymorphic spectrum of the (TG)_*v*_ and (TCTG)_*w*_ tract variability and the occurrence of intergenerational segregation of *CNBP* alleles with uninterrupted CCTG tract. Our data argues in favor of a relative instability of *CNBP* premutated alleles, even if their clinical relevance is still a matter of debate. In this context, the molecular and clinical characterization of additional individuals would be desirable to provide improved genetic testing and counseling for DM2 patients.

## Data Availability Statement

The raw data supporting the conclusions of this article will be made available by the authors, without undue reservation.

## Ethics Statement

The studies involving human participants were reviewed and approved by Ethical Committee PTV Tor Vergata Hospital study # 232/19. The patients/participants provided their written informed consent to participate in this study.

## Author Contributions

AB conceived and designed the study. AB, VVV, MRD’A, and GN drafted and critically revised the manuscript. VVV, LF, and PB collected the data. VVV, LF, IB, PB, MRD’A, and FS handled the genetic testing and molecular characterization of DM2 locus. MB contributed to genetic counselling. RM, GM, GA, AP, and EP contributed to patient’s collection and clinical evaluation. RC and GM contributed to muscle biopsies collection and analysis. All authors contributed to the article and approved the submitted version.

## Conflict of Interest

The authors declare that the research was conducted in the absence of any commercial or financial relationships that could be construed as a potential conflict of interest. The handling editor declared a past co-authorship with one of the authors RM.

## References

[B1] Bachinski L. L. Czernuszewicz T. Ramagli L. S. Suominen T. Shriver M. D. Udd B. (2009). Premutation allele pool in myotonic dystrophy type 2. *Neurology* 72 490–497. 10.1212/01.wnl.0000333665.01888.33 19020295 PMC2677510

[B2] Bachinski L. L. Udd B. Meola G. Sansone V. Bassez G. Eymard B. (2003). Confirmation of the type 2 myotonic dystrophy (CCTG)n expansion mutation in patients with proximal myotonic myopathy/proximal myotonic dystrophy of different European origins: a single shared haplotype indicates an ancestral founder effect. *Am. J. Hum. Genet.* 73 835–848. 10.1086/378566 12970845 PMC1180606

[B3] Binda A. Renna L. V. Bosè F. Brigonzi E. Botta A. Valaperta R. (2018). SCN4A as modifier gene in patients with myotonic dystrophy type 2. *Sci. Rep.* 8:11058. 10.1038/s41598-018-29302-z 30038349 PMC6056531

[B4] Botta A. Bonifazi E. Vallo L. Gennarelli M. Garrè C. Salehi L. (2006). Italian guidelines for molecular analysis in myotonic dystrophies. *Acta Myol.* 25 23–33. 17039977

[B5] Bugiardini E. Rivolta I. Binda A. Soriano Caminero A. Cirillo F. Cinti A. (2015). SCN4A mutation as modifying factor of myotonic dystrophy type 2 phenotype. *Neuromuscul. Disord.* 25 301–307. 10.1016/j.nmd.2015.01.006 25660391

[B6] Cardani R. Baldassa S. Botta A. Rinaldi F. Novelli G. Mancinelli E. (2009). Ribonuclear inclusions and MBNL1 nuclear sequestration do not affect myoblast differentiation but alter gene splicing in myotonic dystrophy type 2. *Neuromuscul. Disord.* 19 335–343. 10.1016/j.nmd.2009.03.002 19345584

[B7] Cardani R. Giagnacovo M. Botta A. Rinaldi F. Morgante A. Udd B. (2012). Co-segregation of DM2 with a recessive CLCN1 mutation in juvenile onset of myotonic dystrophy type 2. *J. Neurol.* 259 2090–2099. 10.1007/s00415-012-6462-1 22407275

[B8] Coenen M. J. H. Tieleman A. A. Schijvenaars M. M. V. A. P. Leferink M. Ranum L. P. W. Scheffer H. (2011). Dutch myotonic dystrophy type 2 patients and a North-African DM2 family carry the common European founder haplotype. *Eur. J. Hum. Genet.* 19 567–570. 10.1038/ejhg.2010.233 21224892 PMC3083617

[B9] Day J. W. Ricker K. Jacobsen J. F. Rasmussen L. J. Dick K. A. Kress W. (2003). Myotonic dystrophy type 2: molecular, diagnostic and clinical spectrum. *Neurology* 60 657–664. 12601109 10.1212/01.wnl.0000054481.84978.f9

[B10] Guo P. Lam S. L. (2016). Short conceptual overview unusual structures of CCTG repeats and their participation in repeat expansion. *Biomol. Concepts* 7 331–340. 10.1515/bmc-2016-0024 27879482

[B11] Hilbert J. E. Ashizawa T. Day J. W. Luebbe E. A. Martens W. B. McDermott M. P. (2013). Diagnostic odyssey of patients with myotonic dystrophy. *J. Neurol.* 260 2497–2504. 10.1007/s00415-013-6993-0 23807151 PMC4162528

[B12] Kamsteeg E. J. Kress W. Catalli C. Hertz J. M. Witsch-Baumgartner M. Buckley M. F. (2012). Best practice guidelines and recommendations on the molecular diagnosis of myotonic dystrophy types 1 and 2. *Eur. J. Hum. Genet.* 20 1203–1208. 10.1038/ejhg.2012.108 22643181 PMC3499739

[B13] Liquori C. L. Ikeda Y. Weatherspoon M. Ricker K. Schoser B. G. H. Dalton J. C. (2003). Myotonic dystrophy type 2: human founder haplotype and evolutionary conservation of the repeat tract. *Am. J. Hum. Genet.* 73 849–862. 10.1086/378720 14505273 PMC1180607

[B14] Liquori C. L. Ricker K. Moseley M. L. Jacobsen J. F. Kress W. Naylor S. L. (2001). Myotonic dystrophy Type 2 caused by a CCTG expansion in intron 1 of ZNF9. *Science* 293 864–867. 10.1126/science.1062125 11486088

[B15] Mahyera A. S. Schneider T. Halliger-Keller B. Schrooten K. Hörner E.-M. Rost S. (2018). Distribution and structure of DM2 repeat tract alleles in the German population. *Front. Neurol.* 9:463. 10.3389/fneur.2018.00463 29973908 PMC6020772

[B16] Matsuura T. Minami N. Arahata H. Ohno K. Abe K. Hayashi Y. K. (2012). Myotonic dystrophy type 2 is rare in the Japanese population. *J. Hum. Genet.* 57 219–220. 10.1038/jhg.2011.152 22258159

[B17] Meola G. Biasini F. Valaperta R. Costa E. Cardani R. (2017). Biomolecular diagnosis of myotonic dystrophy type 2: a challenging approach. *J. Neurol.* 264 1705–1714. 10.1007/s00415-017-8504-1 28550479

[B18] Meola G. Cardani R. (2015). Myotonic dystrophy type 2: an update on clinical aspects, genetic and pathomolecular mechanism. *J. Neuromuscul. Dis.* 2 S59–S71. 10.3233/JND-150088 27858759 PMC5240594

[B19] Meola G. Cardani R. (2017). Myotonic dystrophy type 2 and modifier genes: an update on clinical and pathomolecular aspects. *Neurol. Sci.* 38 535–546. 10.1007/s10072-016-2805-5 28078562

[B20] Montagnese F. Mondello S. Wenninger S. Kress W. Schoser B. (2017). Assessing the influence of age and gender on the phenotype of myotonic dystrophy type 2. *J. Neurol.* 264 2472–2480. 10.1007/s00415-017-8653-2 29086017

[B21] Nakamori M. Sobczak K. Puwanant A. Welle S. Eichinger K. Pandya S. (2013). Splicing biomarkers of disease severity in myotonic dystrophy. *Ann. Neurol.* 74 862–872. 10.1002/ana.23992 23929620 PMC4099006

[B22] Peddareddygari L. R. Grewal A. S. Grewal R. P. (2016). Focal seizures in a patient with myotonic disorder type 2 co-segregating with a chloride voltage-gated channel 1 gene mutation: a case report. *J. Med. Case Rep.* 10:167. 27266866 10.1186/s13256-016-0958-8 PMC4897802

[B23] Pisani V. Panico M. B. Terracciano C. Bonifazi E. Meola G. Novelli G. (2008). Preferential central nucleation of type 2 myofibers is an invariable feature of myotonic dystrophy type 2. *Muscle Nerve* 38 1405–1411. 10.1002/mus.21122 18816606

[B24] Radvanszky J. Surovy M. Polak E. Kadasi L. (2013). Uninterrupted CCTG tracts in the myotonic dystrophy type 2 associated locus. *Neuromuscul. Disord.* 23 591–598. 10.1016/j.nmd.2013.02.013 23561036

[B25] Suominen T. Bachinski L. L. Auvinen S. Hackman P. Baggerly K. A. Angelini C. (2011). Population frequency of myotonic dystrophy: higher than expected frequency of myotonic dystrophy type 2 (DM2) mutation in Finland. *Eur. J. Hum. Genet.* 19 776–782. 10.1038/ejhg.2011.23 21364698 PMC3137497

[B26] Suominen T. Schoser B. Raheem O. Auvinen S. Walter M. Krahe R. (2008). High frequency of co-segregating CLCN1 mutations among myotonic dystrophy type 2 patients from Finland and Germany. *J. Neurol.* 255 1731–1736. 10.1007/s00415-008-0010-z 18807109 PMC4079033

[B27] Thornton C. A. (2014). Myotonic dystrophy. *Neurol. Clin.* 32 705–719. 10.1016/j.ncl.2014.04.011 25037086 PMC4105852

[B28] Turner C. Hilton-Jones D. (2010). The myotonic dystrophies: diagnosis and management. *J. Neurol. Neurosurg. Psychiatry* 81 358–367. 20176601 10.1136/jnnp.2008.158261

[B29] Vanacore N. Rastelli E. Antonini G. Bianchi M. L. E. Botta A. Bucci E. (2016). An age-standardized prevalence estimate and a sex and age distribution of myotonic dystrophy types 1 and 2 in the Rome province, Italy. *Neuroepidemiology* 46 191–197. 10.1159/000444018 26882032

